# Validation of the BARD scoring system for fibrosis detection in nonalcoholic fatty liver disease

**DOI:** 10.6026/973206300213703

**Published:** 2025-10-31

**Authors:** Kamrul Anam, Shahinul Alam, Harun Rashid, Sabbir Hossain, Tanveer Rahman, Nazmul Haque, Rofiqul Islam

**Affiliations:** 1 Department of Gastroenterology, National Gastroliver Institute and Hospital, Mohakhali, Dhaka, Bangladesh, South Asia; 2 Department of Hepatology, Bangladesh Medical University, Dhaka, Bangladesh, South Asia; 3 Department of Hepatology, Shaheed Tajuddin Ahmed Medical College, Gazipur, Bangladesh, South Asia; 4 Department of Hepatology, Dhaka Medical College and Hospital, Dhaka, Bangladesh, South Asia; 5 Department of Hepatology, Rajshahi Medical College and Hospital, Rajshahi, Bangladesh, South Asia; 6 Department of Gastroenterology, 250 Bedded General Hospital Pabna, Bangladesh, South Asia

**Keywords:** Nonalcoholic fatty liver disease (NAFLD), BARD, fibrosis, biopsy

## Abstract

The diagnostic performance of the BARD scoring system in detecting significant fibrosis (≥F2) among Bangladeshi patients with biopsy-proven NAFLD is of interest. A cross-sectional study was conducted on 40 adult NAFLD patients at Bangladesh Medical University, Dhaka. Liver fibrosis was staged histologically from biopsy specimens. The BARD score was calculated for each patient and its diagnostic accuracy was evaluated using sensitivity, specificity, predictive values and ROC curve analysis. Clinical and biochemical predictors of fibrosis were also assessed. Among the cohort, 45% had significant fibrosis (F2-F4). The BARD score demonstrated a sensitivity of 83.3%, specificity of 86.4%, positive predictive value of 83.3%, negative predictive value of 86.4% and an AUROC of 0.908 (p < 0.001). A strong positive correlation was observed between BARD score and fibrosis stage (r = 0.736). Multivariate analysis identified BMI ≥28 kg/m^2^ as an independent predictor of significant fibrosis (OR 17.53, p = 0.022). The BARD score is a reliable noninvasive tool for identifying significant liver fibrosis in Bangladeshi NAFLD patients, facilitating early risk stratification and management in resource-limited settings.

## Background:

Nonalcoholic fatty liver disease (NAFLD) has emerged as the most prevalent chronic liver disorder worldwide, paralleling the global rise in obesity, insulin resistance and type 2 diabetes mellitus [1, 2]. Characterized by excessive hepatic fat accumulation in individuals with minimal or no alcohol consumption, NAFLD encompasses a spectrum ranging from simple steatosis to nonalcoholic steatohepatitis (NASH), which can further progress to varying degrees of fibrosis, cirrhosis, liver failure and hepatocellular carcinoma [3]. The burden of NAFLD is particularly significant in South Asian countries, including Bangladesh, due to the rising prevalence of metabolic syndrome components, such as central obesity and diabetes, often manifesting at lower body mass index (BMI) thresholds than in Western populations [4]. This epidemiological shift is creating an urgent need for effective, accessible strategies to stratify NAFLD patients by fibrosis risk [5, 6-7]. Liver fibrosis is the single most important histological predictor of liver-related and overall mortality in NAFLD, more than inflammation or steatosis [8]. Accurate staging of fibrosis is therefore essential to identify patients at risk for adverse outcomes and to guide treatment strategies [9]. Although liver biopsy remains the gold standard for fibrosis assessment, its invasive nature, potential complications, sampling variability and high cost limit its feasibility for routine use, especially in resource-limited settings [10, 11- 12]. This has driven increasing interest in noninvasive diagnostic approaches that can reliably predict the presence of advanced fibrosis [13, 14].

Several noninvasive scoring models have been proposed to identify patients at risk of significant or advanced fibrosis in NAFLD. Among these, the BARD score-based on three routine clinical parameters: BMI ≥28 kg/m^2^, AST/ALT ratio ≥0.8 and the presence of type 2 diabetes mellitus-has gained attention for its simplicity and ease of implementation [15, 16-17]. The score ranges from 0 to 4 and it were initially shown to have a high negative predictive value (NPV), enabling clinicians to rule out advanced fibrosis with confidence [18, 19-20]. However, subsequent validation studies have demonstrated variability in its diagnostic performance, with relatively low positive predictive value (PPV), particularly across different ethnic groups and clinical settings [21, 22-23]. Given the distinctive metabolic profile and fibrosis risk among South Asian populations and in light of the increasing NAFLD burden in Bangladesh, it is crucial to assess the applicability and accuracy of the BARD score within this population [24, 25- 26]. Current literature lacks sufficient local validation studies of the BARD score in biopsy-proven NAFLD cases, leaving a gap in evidence for its use as a frontline screening tool in this region [27]. Validating the BARD score in a Bangladeshi cohort can inform clinical decision-making, particularly in primary care and hepatology settings where advanced diagnostic modalities may not be readily available [28]. Furthermore, such validation will help to clarify whether region-specific modifications or alternative noninvasive algorithms are needed [29]. The performance of the BARD scoring system in predicting significant liver fibrosis in patients with histologically confirmed nonalcoholic fatty liver disease in a Bangladeshi population [30] is shown. Futher, key clinical and biochemical factors associated with advanced fibrosis, to support risk stratification and optimize patient management in resource-constrained environments [31] is shown. Therefore, it is of interest to validate the BARD scoring system for fibrosis detection in nonalcoholic fatty liver disease.

## Methods:

## Study design and setting:

This cross-sectional study was conducted at the Department of Hepatology, Bangladesh Medical University (BMU), Dhaka, over a two-year period from January 2016 to December 2017. A total of 40 patients with histologically confirmed nonalcoholic fatty liver disease (NAFLD) were included using a non-probability convenience sampling technique.

## Sample size calculation:

A total of 40 patients with biopsy-proven nonalcoholic fatty liver disease (NAFLD) were included in the study. Among them, 18 patients (45%) had significant fibrosis (stage 2-4), while 22 patients (55%) had mild or no fibrosis (stage 0-1). This sample size was sufficient to evaluate the diagnostic performance of the BARD score and to assess the clinical and biochemical predictors of advanced fibrosis in this cohort.

## Patient selection criteria:

Patients were selected based on strict inclusion and exclusion criteria. Adults patients with age between 18 to 65 years with ultrasonographic evidence of fatty liver who gave written informed consent were included in the study. Exclusion criteria encompassed a history of significant alcohol intake-defined as more than 30 grams per day for males and more than 20 grams for females-current or prior use of hepatotoxic medications such as tamoxifen, valproate, amiodarone and methotrexate and any other known liver diseases including hepatitis B, hepatitis C, autoimmune hepatitis, hereditary hemochromatosis, primary biliary cholangitis, primary sclerosing cholangitis, or cirrhosis unrelated to nonalcoholic steatohepatitis (NASH). Pregnant patients or those suffering from liver failure, hypothyroidism, chronic obstructive pulmonary disease (COPD), chronic kidney disease (CKD), or heart failure were also excluded.

## Data collection procedures:

Each participant underwent a comprehensive clinical evaluation, including detailed history-taking and physical examination. Anthropometric measurements such as body mass index (BMI), waist circumference and blood pressure were recorded. Laboratory investigations, conducted at the BMU central laboratory, included complete blood count (CBC), erythrocyte sedimentation rate (ESR), thyroid-stimulating hormone (TSH), fasting blood sugar (FBS), two-hour after-breakfast blood sugar (2HABF), homeostasis model assessment of insulin resistance (HOMA-IR), total and direct bilirubin, alanine aminotransferase (ALT), aspartate aminotransferase (AST), prothrombin time and international normalized ratio (PT-INR), gamma-glutamyl transferase (GGT), serum albumin, hepatitis B surface antigen (HBsAg), anti-HCV antibody, serum ferritin, total cholesterol, high-density lipoprotein (HDL), low-density lipoprotein (LDL) and triglycerides.

## Liver biopsy and fibrosis assessment:

Patients meeting the eligibility criteria were admitted to the hepatology ward and prepared for percutaneous liver biopsy following assessment of coagulation parameters. After a fasting period of 6 to 8 hours, liver biopsies were performed using a 14-gauge Tru-Cut needle under strict aseptic precautions and local anesthesia with 2% lignocaine via the intercostal route, during patient expiration. The liver tissue was preserved in 10% formalin and submitted for histopathological evaluation. Fibrosis staging and NAFLD activity score (NAS) were assessed using the criteria described by Similar study. Fibrosis was staged from F0 to F4, where F0 to F2 was considered mild to moderate and F3 to F4 as advanced fibrosis. Based on histological results, patients were divided into two groups: those with significant fibrosis (F2-F4) and those with no or mild fibrosis (F0-F1). All patients were observed for 24 hours post-biopsy, with no reported complications.

## BARD score calculation:

The BARD score was calculated for each patient using three clinical variables: an AST/ALT ratio of ≥0.8 (2 points), a BMI of ≥28 kg/m^2^ (1 point) and the presence of type 2 diabetes mellitus (1 point). The total score ranged from 0 to 4. As proposed by Harrison *et al.*, a BARD score of 0 or 1 has a high negative predictive value (96%) for advanced fibrosis, whereas a score of 2 or higher is strongly associated with advanced fibrosis (F3-F4), with an odds ratio of 17 [32]. All scores were computed manually and cross-verified using the online calculator at www.pmidcalc.org.

## Data collection, analysis and validation:

Data was collected using structured questionnaires administered during patient visits to the Hepatology Department. Written informed consent was obtained prior to participation. Categorical variables were summarized using frequencies and percentages, while continuous variables were described using means, medians, standard deviations and ranges. Comparisons of clinical and laboratory parameters between fibrosis groups were performed using the chi-square test. BARD score performance was validated using univariate analysis, Spearman's correlation coefficient (r^2^), odds ratio estimation and multivariate logistic regression. Diagnostic accuracy was assessed by calculating sensitivity, specificity, positive predictive value (PPV), negative predictive value (NPV) and area under the receiver operating characteristic (ROC) curve (AUROC). A p-value of less than 0.05 was considered statistically significant. All statistical analyses were performed using SPSS version 24.

## Ethical considerations:

This study was conducted in accordance with the ethical guidelines of the Declaration of Helsinki and Good Clinical Practice (GCP). Participants were thoroughly informed about the objectives, procedures and potential risks and benefits of the study. Written informed consent was obtained from all participants before any procedure, including liver biopsy. Confidentiality was ensured through anonymization using unique identification numbers. Ethical approval was obtained from the Institutional Review Board (IRB) of BMU, Dhaka (Approval No. BMU/2016/3395).

## Results:

The study involved 40 patients (60% female) (Figure 1 - see PDF), with a mean age of 43.3 ± 8.0 years and BMI of 26.8 ± 3.7 kg/m^2^ (65% obese). Waist circumference exceeded metabolic risk in 85%. Hypertension, diabetes and metabolic syndrome were present in 30%, 27.5% and 75%, respectively. Mean fasting glucose was 5.7 ± 3.7 mmol/L; triglycerides 210.4 ± 97.3 mg/dL (60% elevated); and HDL 35.6 ± 8.5 mg/dL (90% low). Elevated liver enzymes were noted in 20-25% of patients. HOMA-IR averaged 2.2 ± 0.4, suggesting insulin resistance in the group (Table 1 - see PDF). Patients with fibrosis scores 2-4 had a significantly higher BMI (28.2 ± 3.2 kg/m^2^ vs. 25.7 ± 3.8 kg/m^2^, p = 0.032) and HOMA-IR (2.7 ± 0.7 vs. 1.9 ± 0.4, p = 0.001), indicating greater obesity and insulin resistance compared to those with fibrosis scores 0-1. The fibrosis 2-4 group also showed significantly higher 2-hour post-load blood glucose levels (9.6 ± 3.3 mmol/L vs. 7.2 ± 3.0 mmol/L, p = 0.021), suggesting impaired glucose tolerance. AST levels were significantly elevated (38.9 ± 25.9 U/L vs. 25.6 ± 12.3 U/L, p = 0.039), as it was reflected at the AST/ALT ratio (0.86 ± 0.28 vs. 0.66 ± 0.17, p = 0.008), both of which are associated with advanced fibrosis. No significant differences were observed between groups in age, blood pressure, lipid profile, GGT, ALP, bilirubin, albumin, ferritin, or platelet count. Overall, obesity, insulin resistance, impaired glucose tolerance, elevated AST and a higher AST/ALT ratio were the most prominent features linked to significant liver fibrosis in this cohort (Table 2 - see PDF). Patients with advanced fibrosis (fibrosis score 2-4) had a significantly higher frequency of lobular inflammation (p = 0.013), with 50% showing grade 2 inflammation compared to only 13.6% in those with mild fibrosis (0-1). A diagnosis of NASH (NAS ≥5) was also significantly more common in the advanced fibrosis group (72.2% vs. 31.8%, p = 0.011). Furthermore, a high BARD score (2-4) was strongly associated with advanced fibrosis (83.3% vs. 13.6%, p = 0.001). In contrast, steatosis grade and hepatocellular ballooning did not differ significantly between fibrosis groups. Collectively, NASH status, lobular inflammation severity and elevated BARD scores emerged as key predictors of advanced fibrosis in this cohort (Table 3 - see PDF). The validity of the BARD score in predicting significant fibrosis was assessed through standard measures of diagnostic performance. The BARD score showed a sensitivity of 83.3% and a specificity of 86.4%, with an overall accuracy of 85.0%. The positive predictive value (PPV) was 83.3%, while the negative predictive value (NPV) was 86.4%. These findings indicate that the BARD score is a reliable non-invasive tool for identifying patients with significant fibrosis in this cohort (Table 4 - see PDF). A positive correlation between the fibrosis score and the BARD score was found in patients with nonalcoholic fatty liver disease. The correlation coefficient (r) was 0.736 and this correlation was statistically significant (p=0.001), indicating that as the BARD score increases, the fibrosis score also tends to increase (Figure 2 - see PDF). Table 5 (see PDF) summarizes the key clinical and biochemical factors associated with significant fibrosis (fibrosis stage 2-4) in this cohort. Patients with advanced fibrosis were significantly more likely to have obesity (BMI ≥25 kg/m^2^, p = 0.027), BMI ≥28 kg/m^2^ (p = 0.001), diabetes mellitus (p = 0.005), a higher AST/ALT ratio (≥0.8, p = 0.001), metabolic syndrome (p = 0.001) and impaired glucose tolerance (IGT) (p = 0.013). Age ≥45 years, HOMA-IR >1.8 and hypertension were not significantly associated with fibrosis stage. Multivariable logistic regression (Table 6 - see PDF) revealed that after adjusting for other variables, only BMI ≥28 kg/m^2^ remained an independent predictor of significant fibrosis (OR 17.53, 95% CI 1.50-100.0, p = 0.022). Diabetes mellitus and AST/ALT ratio ≥0.8 did not retain independent significance, with p-values of 0.394 and 0.999, respectively. The area under the ROC curve (AUC) for the BARD score in predicting fibrosis was 0.908 (95% CI: 0.810 to 1.00), indicating excellent discriminatory power. A cut-off value of 2.0 for the BARD score yielded this sensitivity and specificity values (Figure 3 - see PDF).

## Discussion:

This study sought to validate the diagnostic performance of the BARD scoring system in detecting significant fibrosis (≥F2) among Bangladeshi patients with biopsy-proven nonalcoholic fatty liver disease (NAFLD). Our findings support the BARD score's utility as a noninvasive, cost-effective screening tool with good sensitivity and specificity in identifying patients at risk of advanced fibrosis. Additionally, we examined the predictive capacity of individual clinical parameters and their histological correlates to better understand the pathophysiological basis of fibrosis progression in NAFLD. The BARD score in our study demonstrated a sensitivity of 83.3%, specificity of 86.4% and overall accuracy of 85.0%, with a positive predictive value (PPV) of 83.3% and a negative predictive value (NPV) of 86.4% in detecting significant fibrosis. A statistically significant positive correlation was observed between BARD score and fibrosis stage (r = 0.736, p = 0.001), underscoring its potential as a fibrosis stratification tool. These findings align with previous studies showing the BARD score to be a practical and non-invasive predictor of advanced fibrosis. The original study by Harrison *et al.* (2008) developed the BARD score using BMI ≥28 kg/m^2^, AST/ALT ratio ≥0.8 and presence of diabetes mellitus, reporting an NPV of 96% but a lower PPV of 43%, emphasizing its strength as a rule-out tool [32]. Similarly, Angulo *et al.* emphasized that non-invasive markers like BARD and NAFLD fibrosis scores typically yield high NPVs but relatively modest PPVs, especially in low-prevalence settings [33]. Interestingly, our findings show higher predictive accuracy compared to Harrison's original cohort, possibly due to the higher baseline risk and clustering of metabolic syndrome components among South Asian populations, where insulin resistance, central obesity and type 2 diabetes often occur at lower BMI thresholds. This demographic pattern may partly explain the improved PPV. This interpretation is further supported by data from a recent Indian cohort Shaji *et al.*, which also found high NPVs (>85%) across non-invasive scores, though the BARD score had the lowest AUROC (0.599), reinforcing its primary utility in exclusion rather than confirmation of fibrosis [34].

Additionally, similar performance was noted in Polish and South American cohorts. Cichoz-Lach *et al.* found the BARD score had a PPV of 68.6%, NPV of 96.7% and AUROC of 0.865 for detecting advanced fibrosis in NAFLD patients, while Ruffillo *et al.* reported a BARD AUROC of 0.67 and NPV of 81.3%, further validating its role as an accessible and low-cost screening tool with high exclusion capacity [25, 35]. Multivariate logistic regression identified BMI ≥28 kg/m^2^ as a significant independent predictor of fibrosis (OR = 17.53, p = 0.022), highlighting the pivotal role of sustained adiposity in the pathogenesis of NAFLD and hepatic fibrogenesis. This is in line with findings from Ajmera *et al.* (Framingham Heart Study), which demonstrated a strong association between cumulative overweight exposure-measured as overweight-years-and both NAFLD (aOR = 3.53) and clinically significant fibrosis (aOR = 1.60), independent of age, sex and diabetes (p < 0.001) [36]. These results underscore that not only the degree but also the chronicity of excess weight contributes significantly to hepatic injury via mechanisms involving metabolic stress, lipotoxicity and low-grade inflammation. Although type 2 diabetes and an AST/ALT ratio ≥0.8 were significantly associated with fibrosis in univariate analysis (p = 0.005 and p = 0.001, respectively), they did not retain independent significance in the multivariate model. This attenuation may reflect multicollinearity with BMI or shared pathogenic pathways that limit their standalone predictive value. Nonetheless, other studies including Naskar *et al.* have confirmed that type 2 diabetes remains a critical risk factor for hepatic fibrosis in MASLD, with a reported fibrosis prevalence of 7.4% in diabetic patients based on transient elastography [37]. Their findings further identify elevated systolic blood pressure, serum total bilirubin and AST levels as independent predictors of advanced fibrosis, suggesting a multifactorial interplay in disease progression that goes beyond glycemic control alone. Histological analysis revealed a statistically significant association between advanced fibrosis and lobular inflammation (p = 0.013) and NAS score ≥5 (p = 0.011), consistent with progression from simple steatosis to nonalcoholic steatohepatitis (NASH). These histological features are hallmarks of hepatic inflammation and injury that precede and contribute to fibrosis development [38].

## Conclusion:

We demonstrate that the BARD scoring system is a reliable, noninvasive, and cost-effective tool for detecting significant liver fibrosis in Bangladeshi patients with biopsy-proven nonalcoholic fatty liver disease (NAFLD). The BARD score showed high sensitivity, specificity, and diagnostic accuracy, with a strong correlation to fibrosis stage. These findings support the use of the BARD score in routine clinical practice, especially in resource-limited settings, to prioritize patients for further evaluation or intervention, though comprehensive assessment remains essential for optimal management.

## Disclosure:

The authors have no multiplicity of interest to disclose.

## Financial Disclosure:

The study did not receive any funding.

## Data Availability:

Data availability of this study should be directed to the corresponding author and are available from the corresponding author on reasonable request.
